# Ultra-sensitive measurement of transverse displacements with linear photonic gears

**DOI:** 10.1038/s41467-022-28700-2

**Published:** 2022-02-28

**Authors:** Raouf Barboza, Amin Babazadeh, Lorenzo Marrucci, Filippo Cardano, Corrado de Lisio, Vincenzo D’Ambrosio

**Affiliations:** 1 Dipartimento di Fisica, Università degli studi di Napoli Federico II, Complesso Universitario di Monte S. Angelo, Via Cintia, 80126 Napoli, Italy; 2 CNR-SPIN U.O.S. di Napoli, Via Cintia, 80126 Napoli, Italy

**Keywords:** Imaging and sensing, Optical physics, Optics and photonics

## Abstract

Accurately measuring mechanical displacements is essential for a vast portion of current technologies. Several optical techniques accomplish this task, allowing for non-contact sensing even below the diffraction limit. Here we introduce an optical encoding technique, dubbed “linear photonic gears”, that enables ultra-sensitive measurements of a transverse displacement by mapping it into the polarization rotation of a laser beam. In ordinary ambient conditions, we measure the relative shift between two objects with a resolution of 400 pm. We argue that a resolution of 50 pm should be achievable with existing state-of-the-art technologies. Our single-optical-path scheme is intrinsically stable and it could be implemented as a compact sensor, using cost effective integrated optics. We anticipate it may have a strong impact on both research and industry.

## Abstract (web summary)

The authors introduce linear gears to measure mechanical displacements with high sensitivity. Such gears covert tiny transverse shifts between two birefringent devices into a giant polarization rotation of a laser beam.

## Introduction

Reading out and tracking precisely the position of a system is of key relevance in fields as different as microscopy, mechanical engineering, quantum physics, material science, semiconductor industry, or general relativity^1–5^. To this end, light has emerged as an invaluable tool, as it allows for fast, non-invasive, and accurate sensing^6^. In photonic systems, displacements can be regarded as either parallel or transverse to the main propagation direction of the optical beam. While in the first case triangulation measurements or interferometric setups^7^ can be used, the measurement of transverse displacements (TD) typically relies on the detection of differential current signals from photodiodes^8^. This provides a practical but limited solution in terms of sensitivity and resolution. For improved performance, other techniques are available, which exploit for instance grating interferometry^9^, diffraction-based overlay^10^, or fluorophore imaging^11^.

Structured light^12^, which is an optical field presenting a spatially-varying distribution of amplitude, phase, and/or polarization, emerged recently as a resource in this area^13–20^. By exploiting structured illumination, TD can be indeed measured, for instance, via position-dependent directional scattering from a nanoantenna^13,15,17^ or via centroid tracking of the scattered field distribution^14^. Moreover, by properly sculpting the phase profile of a light beam via a metasurface, an optical ruler exploiting super-oscillations achieves a resolution far below the diffraction limit^16^. Although these methods enable TD measurements with sub-nanometric resolutions, they all rely on high-magnification imaging systems and require one to match light wavelength to specific nano-antenna resonances or to post-process images via reconstruction algorithms. These factors impose limitations in terms of footprint, versatility, cost, and speed, all relevant features of an ideal sensor, besides its sensitivity. Importantly, structured light can be a resource for enhanced sensing purposes even without high-magnification imaging, as for instance in the “photonic gears”, in which a bidirectional mapping between the polarization state and a properly tailored vectorial mode of a paraxial light beam enables a boost of the sensitivity in roll angle measurements^21^.

By combining a similar principle with a Moiré-like sensing scheme^22–24^ here we present and demonstrate an optical encoding method, the *linear photonic gears*, that enables an enhanced sensitivity in TD measurements with a compact, fast, stable, and cost-effective setup.

## Results

### Working principle

**Fig. 1:**
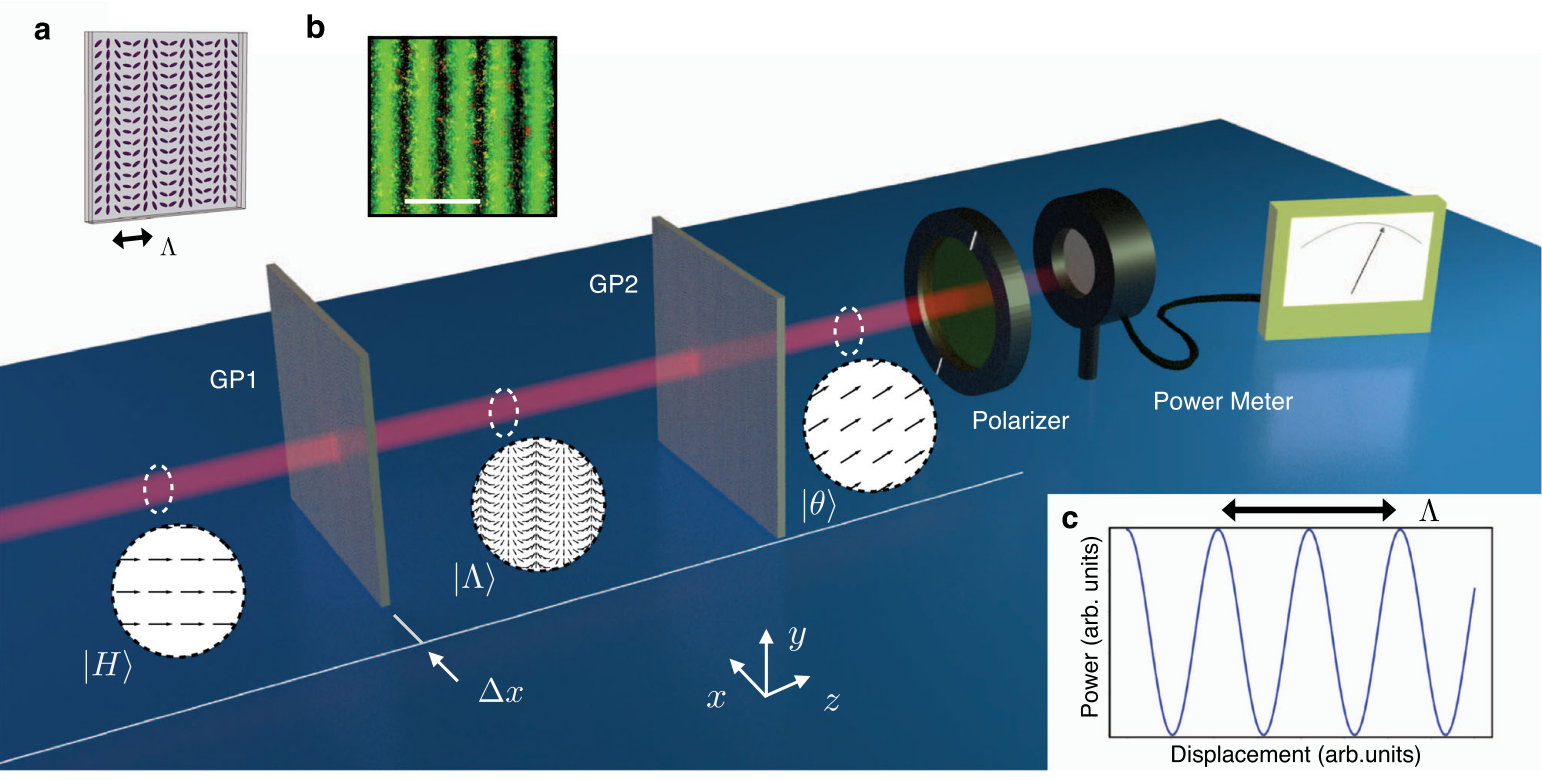
Linear gears concept. A laser beam, linearly polarized along the horizontal direction, is transformed by a *g*-plate (GP1) into a structured light beam $\left|\Lambda \right\rangle$. A second *g*-plate (GP2), identical to GP1, transforms back the polarization mode into a linearly polarized homogeneous one ($\left|\theta \right\rangle$), rotated by an angle Δ*θ* with respect to the input polarization direction. Dashed circles represent the polarization state in three different positions along the laser beam corresponding to $\left|H\right\rangle$, $\left|\Lambda \right\rangle$, and $\left|\theta \right\rangle$, respectively. By monitoring the optical power after a linear polarizer, one can track the transverse displacement Δ*x*. **a** *g*-plate geometry, purple lines represent the orientation of the liquid-crystal molecular director inside the cell. The spatial period is set by the parameter Λ. **b** Micrograph of a *g*-plate with Λ = 50 μm placed between two linear polarizers. The scale bar corresponds to 50 μm. **c** Super-resolving Malus’ law for the linear photonic gear.

Let us consider the situation depicted in Fig. 1 where we are interested in measuring the relative TD Δ*x*, along the *x-*axis, between two objects (GP1 and GP2). To do so we prepare a collimated laser beam in the state $\left|H\right\rangle$, that is uniformly linearly polarized along the horizontal direction, and let it pass through a *g*-plate^25^, lying in the reference frame of GP1. Without loss of generality, we will identify GP1 with the actual *g*-plate in the rest of the paper. A *g*-plate is a patterned liquid crystal slab where the orientation *α* of the molecular director follows the geometry:

1 $$\alpha (x,y)=\frac{\pi }{{{\Lambda }}}x+{\alpha }_{0}$$

where Λ is the spatial period of the device and *α*_0_ is an offset angle (see Fig. 1a). At the exit of the *g*-plate, the optical polarization takes the following expression (see Supplementary Information):

2 $$\left|H\right\rangle \to \left|{{\Lambda }}\right\rangle =\cos [2\alpha (x)]\left|H\right\rangle +\sin [2\alpha (x)]\left|V\right\rangle$$

with $\left|V\right\rangle$ labeling the linear polarization state along the vertical direction. The state $\left|{{\Lambda }}\right\rangle$ represents a structured light beam where the polarization direction varies linearly along the *x*-axis with a period equal to Λ/2. Let us now assume that the beam passes through a second *g*-plate (our second object GP2), identical to the first one but laterally shifted by an amount Δ*x*. As detailed in the Supplementary Information (SI), the output field is then:

3 $$\left|{{\Lambda }}\right\rangle \to \left|\theta \right\rangle =\cos ({{\Delta }}\theta )\left|H\right\rangle +\sin ({{\Delta }}\theta )\left|V\right\rangle$$

that is a homogeneous linearly polarized beam, with the polarization direction rotated by an angle Δ*θ* with respect to the input state and where

4 $${{\Delta }}\theta =\frac{2\pi {{\Delta }}x}{{{\Lambda }}}.$$

Equation (4) represents the *linear gears* mapping between the displacement Δ*x* and the polarization rotation Δ*θ*. Crucially, this rotation can be amplified by reducing the value of the *g*-plates spatial period Λ, as when decreasing the radius of mechanical gears. Let us stress that this holds for any linearly polarized state, and the input $\left|H\right\rangle$ was chosen only for the sake of simplicity. Moreover, in order to neglect the beam diffraction when deriving Eq. (3), we are assuming that the distance *D* between *g*-plates is sufficiently small, that is *D* ≪ *w*_0_Λ/*λ*, where *w*_0_ is the beam radius at the waist position and *λ* is the optical wavelength. As an alternative, for larger values of *D*, a lens system can be used to image the first *g*-plate onto the second one.

To measure Δ*x*, one can read out the optical power after a projection over the initial polarization state, leading to a super-resolving Malus’ law (Fig. 1c)

5 $$P({{\Delta }}x)={P}_{0}{\left|\left\langle H| \theta \right\rangle \right|}^{2}={P}_{0}{\cos }^{2}\left(\frac{2\pi {{\Delta }}x}{{{\Lambda }}}\right)$$

where *P*_0_ is the power of the input laser beam, assuming no power loss in crossing the two *g*-plates. An offset term in the argument of the cosine function (see for details Eq. (S4) in the SI) can be adjusted by rotating either the input polarization of the polarizer orientation. This in turn allows one to accurately shift the Malus’ law curve so as to operate in its linear regions, where the maximum sensitivity *S* is obtained and we get

6 $${P}_{lin}\simeq {P}_{0}\left(\frac{1}{2}\pm \frac{2\pi }{{{\Lambda }}}{{\Delta }}x\right).$$

This corresponds to a sensitivity $S=\left|\frac{dP}{d{{\Delta }}x}\right|=2\pi {P}_{0}/{{\Lambda }}$, that can be increased by reducing Λ. Importantly, as we increase the sensitivity, we concurrently reduce the working range of the linear gears, which is approximately given by Λ/4 (monotonicity interval for the Malus’ law). For bigger displacements, we face, in principle, an ambiguity for the estimation of the correct TD. However, this limit can be overcome in (at least) the following two ways. First, the working point can be always kept in the linear range of the gear by dynamically rotating the polarization analyzer or, equivalently, the input polarization. The degeneracy is then removed by keeping track of this rotation. Second, it is possible to exploit an additional pair of *g*-plates with a period ${{\Lambda }}^{\prime}$ large enough to remove the degeneracy, while the desired resolution is provided by the original *g*-plate pair.

### Experimental implementation

**Fig. 2:**
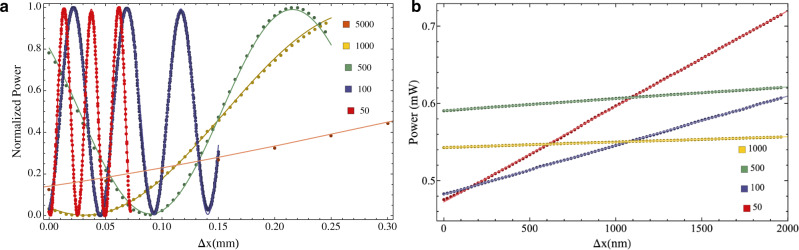
Experimental results. **a** For each value of Λ, we reported the normalized measured power of the horizontal polarization component, as a function of the displacement between the two *g*-plates. The zero displacement point for each curve has been set to allow easy visual comparison between different gears. **b** The same measurement is repeated in the linear part of the calibration curves, with a displacement step of 20 nm. For each configuration, the input optical power was set to a reference value *P*_0_ = 1 mW. In both panels, points represent experimental data, solid lines are best-fit curves according to equations in the main text, experimental points correspond to the average power over 20 independent measurements (error bars, corresponding to standard deviations, are smaller than the data point size). Specific colors label datasets that are associated with different spatial periods Λ (expressed in μm in the legend).

To validate our findings, we realized the setup sketched in Fig. 1 (more details in SI). Specifically, we sent a collimated He:Ne laser beam (*λ* = 633 nm, horizontally polarized) through a pair of *g*-plates, considering in our experiment five different types of pairs with spatial periods Λ = 5000, 1000, 500, 100, 50 μm, respectively. At the exit of the second plate, a polarizer was used to filter the desired linear polarization component and the optical power was measured by a power meter. To displace the plates in a controllable manner, the second cell was mounted on a motorized translation stage. Normalized measured powers for different TDs are reported in Fig. 2a, together with the best-fit curves according to Eq. (5). Obtained data nicely reproduce the expected oscillatory behavior.

**Fig. 3:**
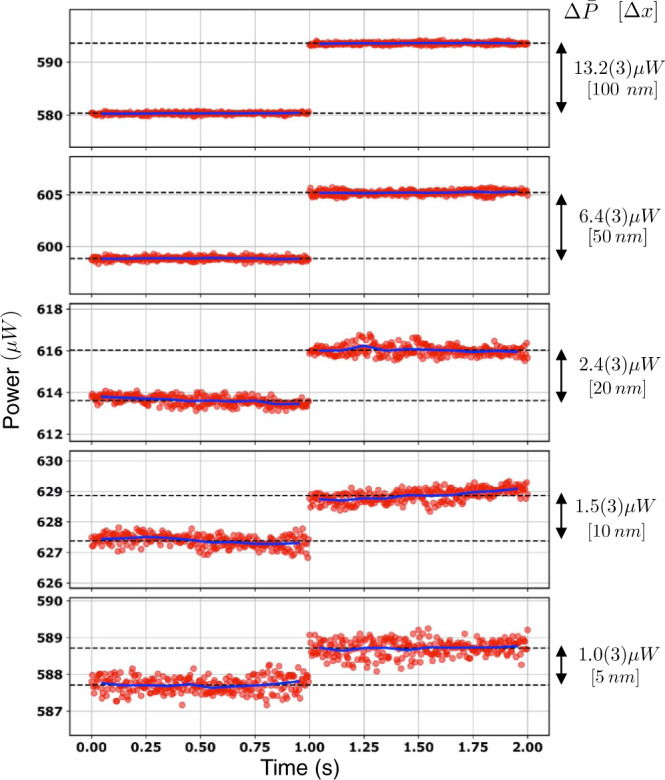
Step measurements for gear Λ = 50 μm. Each plot reports the optical power measured for 1 s (250 red points) before and after a controlled step Δ*x* of the translation stage. Blue lines represent the average power calculated over time intervals of 0.1 s while black dashed lines mark the total average power calculated before and after each step. The corresponding power difference ${{\Delta }}\bar{P}$ is reported, together with the step amplitude (in square brackets), on the right side of each plot.

To evaluate and compare the sensitivity of different gears, we then focused our measurements on the linear region. We, therefore, set the optical power to a reference value *P*_0_ = 1 mW and measured the calibration curve *P*(Δ*x*) in the linear region, for a total displacement of 2 μm with steps of 20 nm (see Fig. 2b). To improve the plot clearness, each dataset has been measured in a slightly shifted region with respect to the middle of the fringe (but still abundantly in the linear region of the calibration curve). A comparison between the slopes of the curves clearly shows the advantage in terms of sensitivity that is obtained by decreasing Λ. This can be readily quantified by performing a linear fit for each curve. The best sensitivity *S* = 124.0 ± 0.1 nW/nm was obtained, as expected, for *g*-plates with Λ = 50 μm. We, therefore, evaluated the actual resolution of our detection system for this configuration. To this end, we repeatedly measured the optical power for a time interval of 1 s (250 points), before and after a controlled step of the translation stage. We report these results in Fig. 3, where the red dots represent experimental data, while black dashed lines mark the average power as calculated over each set of 250 points. We started with a “large" displacement Δ*x* = 100 nm, which led to a mean power difference ${{\Delta }}\bar{P}=13.2$ μW, and gradually decreased the step size. The smallest measured displacement Δ*x* = 5 nm, corresponding to approximatively *λ*/125, is still clearly resolved as the measured ${{\Delta }}\bar{P}=1.0$ μW is significantly larger than its error bar (*σ* = 0.3 μW), calculated as the quadrature sum of the standard deviations of the power distribution before and after the step, respectively. Further reducing the displacement would not lead to meaningful results, as the nominal displacement accuracy of our translation stage is approximately 2 nm. Let us notice that the power standard deviation when using *g*-plates with Λ = 50 μm was typically *σ*_*P*_ = 0.2 μW, yet it reduced to 0.1 μW when the plates were switched off. Therefore, we ascribe the residual value of the standard deviation of our signal to vibrations of the optomechanical components.

An estimate of the system resolution is obtained in terms of the ratio *R* = *σ*/*S*. To evaluate this parameter, it is crucial to get rid of fast signal oscillations that are mainly due to mechanical fluctuations of our system. To this end, we considered the average power over time intervals of 0.1 s (blue lines in Fig. 3), yielding a typical standard deviation *σ*_*P*_ = 0.05 μW. This corresponds to a sub-nanometric resolution *R* ≃ 400 pm $\left(\lambda /1580\right)$.

In the case of an ideal target without vibrations, this result could be further improved if fluctuations in optical power are minimized, for instance by employing an ultrastable laser or a balanced detector. The value of Λ, on the other hand, can be decreased down to a few microns (or even below if replacing *g*-plates with dielectric metasurfaces^26^). By referring to state-of-the-art liquid crystal technology, we can consider *g*-plates with Λ = 6 μm^27^ which, if considering the power fluctuations of our system (at *P*_0_ = 1 mW) would yield the remarkable resolution of *R* ≃ 50 pm, corresponding to approximatively *λ*/12,500.

## Discussion

In conclusion, in this paper, we have reported an optical encoding technique that enables ultra-sensitive measurements of TDs. This is done by directly mapping displacements between two objects into polarization rotations of a collimated laser beam. Let us note that, in principle, it is possible to encode TD in modulations of the field amplitude^24^ (rather than polarization), by using a pair of amplitude masks instead of *g*-plates. However, as detailed in SI, linear gears outperform amplitude encoding both in terms of sensitivity (by almost an order of magnitude) and stability. By operating in ambient conditions and room temperature with a He-Ne laser, low optical power, and a standard power meter, we demonstrated a resolution of *R* = 400 pm, which could plausibly be brought down to *R* = 50 pm by considering existing state-of-the-art liquid crystal fabrication technology. Although our results are for one-dimensional displacements, they can be easily extended to a generic displacement in the transverse plane. A single gear works in one direction only, which is uniquely determined by the modulation axis of the *g*-plate pair (the *x*-axis in our experiment). The measurement process is insensitive to movements in the orthogonal direction. As a result, one could measure arbitrary displacements in the transverse plane by combining two orthogonal linear gears. Such a scheme would require a second laser beam passing through another pair of *g*-plates with a grating vector along *y* and a separate detection channel.

Let us finally remark that this result is obtained with a practical setup that relies on a single optical path, thus not suffering from the typical instabilities that affect metrological interferometric instruments. Our scheme could be eventually implemented in a compact sensor with cost-effective integrated laser diodes and photodetectors. We envisage the use of this technology, for both research and industry, in a number of future sensing devices, for instance for monitoring deformations and displacement of precision components or structures, measuring material properties such as elasticity modulus, in nanofabrication or microscopy, with *g*-plates mounted or directly fabricated on the objects to be tracked.

## Supplementary information

Supplementary Information

## Data Availability

The data that support the findings of this study are available from the corresponding authors upon reasonable request.
