# Supplementary material for: Ultra-sensitive measurement of transverse displacements with linear photonic gears
Source: Nat Commun. 2022 Feb 28;13:1080. doi: 10.1038/s41467-022-28700-2 (PMC8885676; doi:10.1038/s41467-022-28700-2)
Supplement: Supplementary file 1 — Supplementary Information [file 41467_2022_28700_MOESM1_ESM.pdf]

Supplementary Information: Ultra-sensitive measurement of transverse displacements with linear photonic gears

Raouf Barboza,[1] Amin Babazadeh,[1] Lorenzo Marrucci,[1] Filippo Cardano,[1,*] Corrado de Lisio,[1,2] and Vincenzo D'Ambrosio[1,†]

[1]*Dipartimento di Fisica, Università di Napoli Federico II, Complesso Universitario di Monte S. Angelo, Via Cintia, 80126 Napoli, Italy*

[2]*CNR-SPIN U.O.S. di Napoli, Via Cintia, 80126 Napoli, Italy*

S1. FABRICATION OF g-PLATES

g-plates are fabricated using a well established procedure detailed in the following. Two ITO (indium tin oxide) coated glass slabs ($25 \times 25\text{mm}^2$) are spin coated with a solution of Brilliant Yellow, a commercial azo dye in N,N-Dimethylformamide. Spacer beads with diameter 6μm are deposited on one of the substrates (ITO side), then the two glass slabs are glued together (ITO sides inward) to stably maintain the 6μm spacing gap. The desired anchoring pattern is written onto the photo-alignment layer via 1D direct laser writing. The liquid crystal cell is then filled with a nematic liquid crystal E7. Finally, electric contact are added (ITO side) in order to provide control on the effective extraordinary refractive index. At moderate bias voltages, the g-plate behave effectively like an electrically tunable polarization grating.

S2. WORKING PRINCIPLE DETAILS

The action of a g-plate is determined by its birefringent optical retardation δ, whose value can be adjusted by tuning an external alternating voltage [1]. By setting $\delta = \pi$, the g-plate can be described as a geometric-phase grating whose action on the polarization state can be easily written in the circular polarization basis as:

$$|L/R\rangle \rightarrow e^{\pm i2\alpha(x)} |R/L\rangle \tag{S1}$$

where $|L\rangle$ and $|R\rangle$ stand for left and right circular polarization states respectively and $\alpha(x)$ is a linear function of the transverse position given by Equation (1) of the main text. Let us now consider a Gaussian beam $|H\rangle$, uniformly polarized along the horizontal direction (x axis), and propagating along the z axis. The g-plate transformation on the input beam results in:

$$|H\rangle \rightarrow |\Lambda\rangle = \frac{1}{\sqrt{2}} \left(e^{i2(\pi x/\Lambda + \alpha_0)} |R\rangle + e^{-i2(\pi x/\Lambda+\alpha_0)} |L\rangle \right) \tag{S2}$$

Essentially, each circular polarization component is a Gaussian beam, propagating along a direction in the xz plane forming with the z axis an angle that is approximately given by $\simeq \pm\lambda/\Lambda$. As such, in the near field the beam keeps its Gaussian envelope, yet it features a spatially-inhomogeneous polarization pattern (see Fig. 1 in the main text). After passing through a second g-plate, identical to the first one but laterally displaced by an amount Δx, the optical field is described by a state:

$$|H\rangle \rightarrow |\theta\rangle = \frac{1}{\sqrt{2}} \left(e^{i\Delta\theta} |R\rangle + e^{-i\Delta\theta} |L\rangle \right) = \cos(\Delta\theta) |H\rangle + \sin(\Delta\theta) |V\rangle \tag{S3}$$

with $\Delta\theta = 2\pi\Delta x/\Lambda$

* filippo.cardano2@unina.it
† vincenzo.dambrosio@unina.it

S3. EXPERIMENTAL DETAILS

To validate our predictions we implemented the linear gear with the experimental setup depicted in Figure (1). A collimated He-Ne laser beam is initialized in the $|H\rangle$ state by a linear polarizer (Pol1) while a half wave plate (HWP1), placed before Pol1, is used to control the optical power P_0. The linear gear is then implemented via two identical g-plates (GP1 and GP2). In order to control the transverse displacement Δx between the two devices, GP2 is mounted on a motorized translation stage. Depending on the maximum required TD, we used two different translation stages, one with a position accuracy of 100 nm and long travel range, and a second one, based on a piezoelectric positioner, with a displacement accuracy of 2 nm. The power P(Δx) is recorded by a power meter (PM) placed after a second polarizer (Pol2) and a spatial filter (Lens + Iris in the focal plane). The latter is used to improve the Malus' law visibility, as it filters out unwanted light components associated with high spatial frequencies (possibly due to inaccuracies in the tuning of g-plates or in their pattern). A second half waveplate (HWP2) is placed between Pol2 and GP2 to rotate the analysed polarization direction, in order to set the working point in the desired position.

Finally, let us notice that in order to properly take into account experimental imperfections (mainly polarization purity, g-plate conversion efficiency and background noise) equations 5 and 6 need to be slightly modified by adding an extra offset term P_{off}. This term has no direct effect on the sensitivity as we consider $P_0 = P_{\text{tot}} - P_{\text{off}}$, where P_{tot} is the total power of the beam reaching the power meter, therefore we omitted it in the main text for the sake of clarity.

S4. PERFORMANCE

Here, we derive a comparative performance analysis of linear optical encoders demonstrating the advantage of the linear photonic gears with respect to encoders based on amplitude gratings. For the sake of simplicity, we limit our discussion to sine and binary amplitude gratings, by considering simple models, able to give the contour for both quantitative and qualitative results, without loss of generality.

We assume an input Gaussian beam with power P_0, waist ω, centered at x_0, i.e. $I(x) = P_0\sqrt{\frac{2}{\pi\omega^2}}e^{-2(x-x_0)^2/\omega^2}$. As in the main section, we assume that the two gratings are close enough in order to neglect diffraction effects. A single g-plate is effectively an electrically controlled birefringent plate with varying optical axis. As such, it can be modeled using Jones matrices (written in left/right handed reference). Here,

$$J(\delta,\theta) = \begin{pmatrix} \cos\frac{\delta}{2} & i\sin\frac{\delta}{2}\ e^{-2i\theta} \\ i\sin\frac{\delta}{2}\ e^{2i\theta} & \cos\frac{\delta}{2} \end{pmatrix},$$

with θ the orientation of the optical axis, and δ the effective phase shift between slow and fast axis. For half-wave tuned g-plates, $\delta = \pi$, the Jones matrix of the stacked g-plated pairs reads as:

$$T(\theta_2,\theta_1) = J(\pi,\theta_2)\cdot J(\pi,\theta_1) = -\begin{pmatrix} e^{2i(\theta_1-\theta_2)} & 0 \\ 0 & e^{-2i(\theta_1-\theta_2)} \end{pmatrix}.$$

When the second g-plate is shifted about Δx, having written $\theta_2(x) = \theta(x-x_2-\Delta x)$, $\theta_1(x) = \theta(x-x_1)$, where x_1 and x_2 represent constant offsets, with $\theta(x) = \pi x/\Lambda$, the effective transmission coefficient of a linearly polarized beam, horizontally (without loss of generality), analyzed through a polarizer at angle p reads as:

$$T(\Delta x) = \cos^2(2\pi\Delta x/\Lambda + 2\pi(x_2-x_1)/\Lambda + p). \tag{S4}$$

The analyzer orientation can therefore be used to shift the operating point. We then set $T(\Delta x) = \cos^2(2\pi\Delta x/\Lambda)$. Interestingly the effective transmission coefficient is constant across the exit plane thus the recorded power is simply

$$P(\Delta x)/P_0 = \cos^2(2\pi\Delta x/\Lambda), \tag{S5}$$

independently from the center and waist, generally from amplitude profile of the input beam.

For sine grating pairs with transmission coefficient $T(x) = (1+\cos(2\pi x/\Lambda))/2$, the recorded intensity for a lateral shift Δx is given by:

$$\begin{aligned} P(\Delta x)/P_0 \ &= \tfrac{1}{4} + \tfrac{1}{8}\cos\left(\tfrac{2\pi}{\Lambda}\Delta x\right) + \tfrac{1}{4}\left[\cos\left(\tfrac{2\pi}{\Lambda}x_0\right) + \cos\left(\tfrac{2\pi}{\Lambda}(\Delta x - x_0)\right)\right]e^{-\frac{1}{2}(\pi\omega/\Lambda)^2} \\ &\quad + \tfrac{1}{8}\cos\left(\tfrac{2\pi}{\Lambda}(\Delta x - 2x_0)\right)e^{-2(\pi\omega/\Lambda)^2}. \end{aligned} \tag{S6}$$

For waists larger than the grating period,

$$P(\Delta x)/P_0 = \frac{1}{4} + \frac{1}{8}\cos\left(2\pi\Delta x/\Lambda\right). \tag{S7}$$

For binary gratings pairs with duty cycle 1/2 (maximum range and sensitivity) centered in 0, the recorded output power for lateral displacement $|\Delta x| \leq \Lambda/2$ reads as:

$$P(\Delta x)/P_0 = \begin{cases} \frac{1}{2}\sum_{n=-\infty}^{\infty}\left[\mathrm{erf}\left(\frac{\sqrt{2}}{\omega}(\Lambda/4 + n\Lambda - x_0)\right) - \mathrm{erf}\left(\frac{\sqrt{2}}{\omega}(-\Lambda/4 + n\Lambda - x_0 + \Delta x)\right)\right], \\ 0 \leq \Delta x \leq \Lambda/2 \\ \frac{1}{2}\sum_{n=-\infty}^{\infty}\left[\mathrm{erf}\left(\frac{\sqrt{2}}{\omega}(\Lambda/4 + n\Lambda - x_0 + \Delta x)\right) - \mathrm{erf}\left(\frac{\sqrt{2}}{\omega}(-\Lambda/4 + n\Lambda - x_0)\right)\right], \\ -\Lambda/2 \leq \Delta x \leq 0. \end{cases} \tag{S8}$$

For waists larger than the grating spacing, we have:

$$P(\Delta x)/P_0 = 1/2 - |\Delta x|/\Lambda. \tag{S9}$$

From the results above, one can easily grasp the advantages of g-plates over these amplitude masks. In the optimal case, when the waist is larger than the grating period, the linear sensitivity of the sine encoder is $S_{\mathrm{sine}} = P_0\pi/(4\Lambda)$, for the binary $S_{\mathrm{binary}} = P_0/\Lambda$. Therefore the linear gears offers a factor 8 and 2π of improvement in sensitivity over sine and binary gratings, respectively.

Moreover, for smaller values of the beam waist ($\omega < \Lambda/2$), the recorded intensity depends on the beam position for the case of amplitude gratings. As a consequence, factors such pointing instabilities or intensity distribution fluctuations in the beam profile, even for typical dimensions smaller than the grating period, could be particularly detrimental in the case of amplitude grating encoders. Intensity distribution fluctuation are particularly relevant in environments (i.e. high precision machining) where dust particles might scatter the laser beam causing position readout errors. Linear photonic gears, on the other hand, are immune to these effects since the recorded intensity (in the case of perfectly tuned g-plates) neither depend on the laser beam width nor its position.

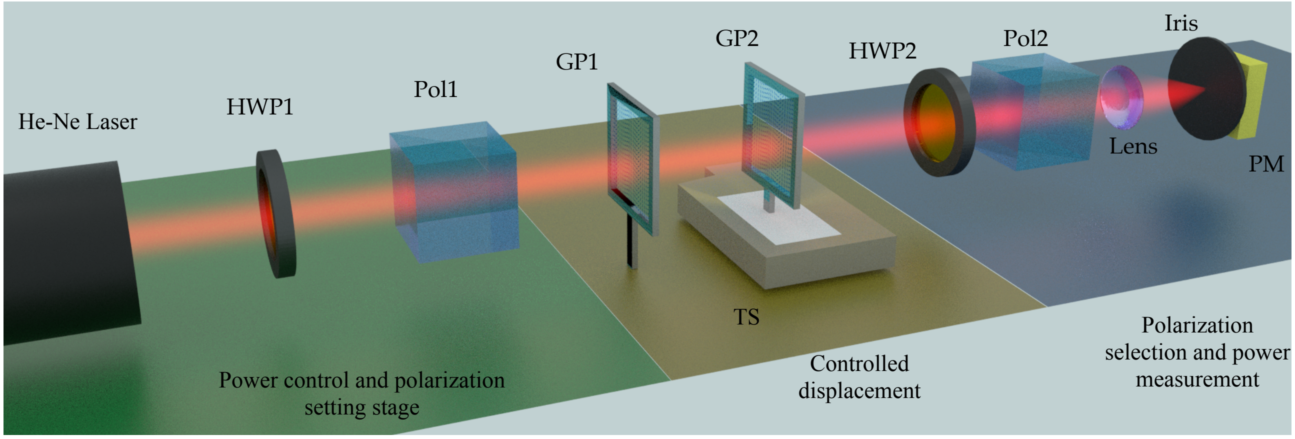

Supplementary Figure 1. Detailed experimental setup: A collimated He-Ne laser beam, is controlled in intensity and set into horizontal polarization state via a half waveplate (HWP1) and a polarizer (Pol1). The linear gear is implemented with the two g-plates (GP1 and GP2). The displacement between the two devices is controlled via a motorized translation stage (TS). A second pair of half waweplate (HWP2) and polarizer (Pol2) allows one to project the polarization along an arbitrary linear state. By rotating HWP2 it is possible to set the working point. The optical power is measured via a power meter (PM) after a spatial filter (lens + iris) that blocks the unconverted light thus increasing the overall fringe visibility.

[1] Piccirillo, B., D'Ambrosio, V., Slussarenko, S., Marrucci, L. & Santamato, E. Photon spin-to-orbital angular momentum conversion via an electrically tunable q-plate. *Applied Physics Letters* **97**, 241104 (2010).
